# First Molecular Detection and Genetic Characterization of Porcine Circovirus 5 in Diagnostic Swine Samples from China

**DOI:** 10.3390/vetsci13070614

**Published:** 2026-06-25

**Authors:** Jia-Qi Zhang, Jia-Xin Li, Hui-Lin Qu, Yu-Jie Miao, Xi-Meng Chen, Lan-Lan Zheng, Yi-Lei Li, Hong-Ying Chen, Shi-Jie Ma

**Affiliations:** 1 College of Veterinary Medicine, Henan Agricultural University, Zhengdong New District Longzi Lake 15#, Zhengzhou 450046, China; 19503633319@163.com (J.-Q.Z.); 18348239448@163.com (H.-L.Q.); 15136369362@163.com (Y.-J.M.); 15680836015@163.com (X.-M.C.); zhll2000@sohu.com (L.-L.Z.); 2 Faculty of Science, University of Melbourne, Parkville, VIC 3010, Australia; cynthia.yunlian@gmail.com; 3 Ministry of Education Key Laboratory for Animal Pathogens and Biosafety, College of Veterinary Medicine, Henan Agricultural University, Zhengzhou 450046, China; 4 Henan Province Key Laboratory for Animal Food Pathogens Surveillance, College of Veterinary Medicine, Henan Agricultural University, Zhengzhou 450046, China

**Keywords:** porcine circovirus type 5, molecular detection, genomic characterization, phylogenetic analysis, co-detection, swine

## Abstract

Porcine circovirus type 5 (PCV5) is a recently reported porcine-associated CRESS DNA virus, but information regarding its occurrence, genomic characteristics, and evolutionary relationship remains limited. In this study, a total of 100 diagnostic samples collected from clinically diseased pigs from 27 commercial swine farms in 16 cities across seven provinces of China during 2025 were screened for PCV5 using quantitative PCR. PCV5 was detected in 22% (22/100) of the tested samples, with positive samples identified in Henan and Fujian provinces among the sampled regions. PCV5-positive samples were mainly fecal samples and were obtained from pigs showing diarrhea, respiratory signs, wasting, or systemic disease. Co-detection analysis showed that most PCV5-positive samples were also positive for other swine viral pathogens, particularly PCV3, PCV2, and PEDV, indicating that the clinical significance of PCV5 should be interpreted cautiously. Complete genome amplification and sequencing yielded two identical PCV5 genomes from Henan and Fujian provinces. A representative strain, designated PCV5-Henan2025-ZJQ01, was further characterized and deposited in GenBank under accession number PZ496079. The complete genome was 2903 nt in length and contained a positive-sense ORF encoding Cap and a negative-sense ORF encoding Rep, showing a distinct genomic organization compared with classical porcine circoviruses. Phylogenetic analysis based on Rep and Cap amino acid sequences showed that PCV5-Henan2025-ZJQ01 was closely related to previously reported PCV5-related sequences but distinct from classical PCV1–PCV4. These findings provide additional molecular and genomic evidence for PCV5 in Chinese swine diagnostic samples and support the need for continued surveillance and further studies on its epidemiological and pathogenic significance.

## Simple Summary

Porcine circovirus type 5 (PCV5) is a newly reported virus detected in pigs, but information about its distribution and genetic characteristics is still limited. In this study, we tested 100 diagnostic samples collected from clinically diseased pigs from seven provinces in China during 2025. PCV5 was detected in 22 samples, mainly from fecal samples, and PCV5-positive samples were found in Henan and Fujian provinces among the sampled regions. Two complete PCV5 genome sequences were obtained, and the representative strain PCV5-Henan2025-ZJQ01 was further analyzed. Genomic and phylogenetic analyses showed that this strain was closely related to previously reported PCV5 sequences but differed from classical porcine circoviruses in several genomic features. Most PCV5-positive samples were also positive for other swine viral pathogens, indicating that the clinical importance of PCV5 should be interpreted cautiously. These findings provide additional molecular evidence for PCV5 in Chinese swine diagnostic samples and may help guide future surveillance and research on this emerging virus.

## 1. Introduction

Eukaryotic circular Rep-encoding single-stranded DNA viruses (CRESS DNA viruses) are a diverse group of small circular DNA viruses characterized by compact genomes, replication-associated proteins (Rep), and broad host associations [1,2]. Among these viruses, members of the family *Circoviridae* are small, non-enveloped viruses with circular single-stranded DNA genomes. According to the International Committee on Taxonomy of Viruses (ICTV), the family *Circoviridae* comprises two genera, *Circovirus* and *Cyclovirus* [3]. Members of *Circoviridae* generally encode at least two major proteins, the capsid protein (Cap) and Rep, which are usually arranged in an ambisense orientation [4]. In classical *Circoviruses*, the intergenic region between the major open reading frames (ORFs) contains a stem-loop structure with a conserved nonanucleotide motif, which serves as the origin of rolling-circle replication.

Porcine circoviruses (PCVs) are among the smallest known animal viruses and have attracted considerable attention because of their economic and veterinary importance [5,6]. Currently, five types of PCV have been reported, namely PCV1, PCV2, PCV3, PCV4, and newly identified PCV5 [7]. PCV1 is generally considered nonpathogenic, whereas PCV2 is the major causative agent associated with a broad spectrum of porcine circovirus-associated diseases (PCVAD), including PCV2 systemic disease (PCV2-SD), PCV2 subclinical infection (PCV2-SI), PCV2 reproductive disease (PCV2-RD), and porcine dermatitis and nephropathy syndrome (PDNS), resulting in economic losses in major swine-producing countries [8,9]. PCV3 was originally reported in 2015 in swine farms in the United States [10,11] with cardiac and multisystemic inflammation, reproductive failure, PDNS-like disease, and respiratory or enteric disorders [10,11,12,13,14]. PCV4 was subsequently identified in pigs with clinical signs such as respiratory disease, diarrhea, and PDNS-like lesions in China in 2019 [15], and has since been detected in several countries, including South Korea, Malaysia, Thailand, and European countries [16,17,18,19,20,21]. However, compared with PCV2, the pathogenic roles of PCV3 and PCV4 remain less clearly defined.

Recently, a novel porcine-associated CRESS DNA virus, tentatively named porcine circovirus type 5 (PCV5), was reported in pigs with respiratory, diarrheal, and reproductive disorders in China [22]. The initial study provided important evidence for the existence of PCV5, including viral DNA detection in clinical samples, analysis of tissue distribution, histological examination, virus isolation, electron microscopic visualization, and serological detection [22]. These findings suggested that PCV5 may have potential clinical relevance in swine populations. Nevertheless, as with many newly identified CRESS DNA viruses, detection in diseased animals does not by itself establish a direct causal relationship with clinical disease. Further molecular, epidemiological, and experimental studies are therefore required to clarify the distribution, genetic diversity, evolutionary relationship, and pathogenic significance of PCV5.

Molecular characterization is particularly important for newly emerging circovirus-like viruses because these viruses may differ substantially in genome size, ORF orientation, conserved replication motifs, and phylogenetic position. For PCV5, available genomic information remains limited, and its relationship with classical porcine circoviruses and other related CRESS DNA viruses has not been fully clarified. In addition, field samples from diseased pigs frequently contain multiple viral pathogens, which complicates the interpretation of clinical signs associated with PCV5 detection. Therefore, studies that combine diagnostic screening, complete genome sequencing, co-detection analysis, and phylogenetic comparison are needed to provide a more reliable molecular basis for future surveillance and pathogenicity studies.

In the present study, clinical samples collected from swine farms in seven provinces of China during 2025 were screened for PCV5 using quantitative PCR. PCV5-positive samples were further subjected to complete genome amplification, sequencing, genomic characterization, and phylogenetic analysis based on Rep and Cap amino acid sequences. In addition, co-detection of common swine viral pathogens was analyzed to provide context for interpreting the clinical background of PCV5-positive samples. This study aimed to provide additional molecular and genomic evidence for PCV5 in Chinese swine diagnostic samples and to improve current understanding of the genetic characteristics of this newly reported porcine-associated CRESS DNA virus.

## 2. Materials and Methods

### 2.1. Sample Collection and Preparation of Viral Nucleic Acids

A total of 100 diagnostic samples originating from 100 individual clinically diseased pigs were submitted to our laboratory during routine disease investigations. The samples were collected from 27 commercial swine farms in 16 cities across seven provinces of China, including Henan, Hunan, Shanxi, Zhejiang, Jiangxi, Anhui, and Fujian, from February to December 2025. The sampled pigs included newborn piglets (*n* = 9), suckling piglets (*n* = 5), weaning pigs (*n* = 59), growers (*n* = 12), and grow-finishing pigs (*n* = 15). The sample types included feces (*n* = 29), intestine (*n* = 36), liver (*n* = 5), spleen (*n* = 3), lung (*n* = 1), blood (*n* = 19), and rectal swabs (*n* = 7). Fecal samples, rectal swabs, and blood samples were collected from live pigs during routine clinical investigations, whereas tissue samples (intestine, liver, spleen, and lung) were collected during post-mortem examination of pigs that had died or been euthanized because of severe clinical disease. The observed clinical signs included diarrhea, systemic signs, wasting, and respiratory signs, including dyspnea, cough, and mild tachypnea. Each sample, including each organ specimen, was processed and tested individually, and no pooled samples were used. Because the samples were submitted during routine diagnostic investigations rather than collected through a randomized prevalence survey, they were regarded as diagnostic convenience samples from clinically diseased pigs.

All samples were stored at −80 °C in the Key Laboratory for Animal-derived Food Safety of Henan Province, China. For tissue samples, approximately 0.3 g of each tissue was homogenized in 1 mL of phosphate-buffered saline (PBS, pH 7.4) to prepare a tissue suspension. The resulting suspensions were subjected to three freeze–thaw cycles, followed by centrifugation at 12,000× *g* for 5 min at 4 °C to obtain clarified supernatants. Viral DNA/RNA was extracted using the TaKaRa MiniBEST Viral RNA/DNA Extraction Kit Ver.5.0 (Takara, Dalian, China), and then viral RNA was reverse transcribed into cDNA using the TIANScriptII RT Kit (TIANGEN, Beijing, China). The DNA and cDNA were stored at −80 °C until further use.

### 2.2. Detection of PCV5

PCV5 was detected using a SYBR Green I-based real-time PCR (qPCR) assay, as described previously [22]. Briefly, the 20-μL reaction mixture consisted of 10 μL of 2× ChamQ Universal SYBR qPCR Master Mix (Vazyme, Nanjing, China), 0.5 μL (25 μM) of each primer (Table S1), 2 μL of template DNA, and 7 μL of ddH_2_O. The qPCR assay was conducted on a CFX96™ system (Bio-Rad Inc., Hercules, CA, USA) under the following conditions: 2 min at 95 °C, and then 40 cycles of 10 s at 95 °C, 20 s at 60 °C. Each qPCR run included nuclease-free water as a negative control to monitor reagent contamination. No dedicated internal or external extraction controls were included in this retrospective diagnostic screening. The Cq values obtained from PCV5-positive samples were recorded and used to guide the selection of samples for subsequent complete genome amplification.

Meanwhile, other common viruses, including porcine epidemic diarrhea virus (PEDV), porcine deltacoronavirus (PDCoV) [23] and porcine reproductive and respiratory syndrome virus (PRRSV) [24] were also detected using RT-qPCR assay based on SYBR Green I as previously described. Porcine circovirus 2 (PCV2), porcine circovirus 3 (PCV3) and porcine circovirus 4 (PCV4) were also detected using a real-time PCR assay based on Taqman probe as previously described [25]. All co-detection analyses presented in this study were performed at the sample level rather than at the animal or farm level.

### 2.3. Genome Amplification, Sequencing and Genomic Analysis of PCV5

Complete genome sequencing requires successful amplification across multiple overlapping fragments, and a positive qPCR result does not necessarily guarantee recovery of the full viral genome. To maximize the likelihood of obtaining complete viral genomes, four samples with relatively low Cq values were selected for genome amplification and sequencing. Complete genome amplification was performed using three pairs of overlapping primers (Table S2) as described previously [22]. PCR was carried out in a 25-μL reaction mixture: 12.5 μL of KOD One™ PCR Master Mix (Toyobo, Shanghai, China), 1.5 μL of template DNA, 0.5 μL of each primer (25 μM) and an appropriate amount of ddH_2_O. Cycling conditions were 94 °C for 5 min, 39 cycles of 98 °C for 10 s, 58 °C for 5 s, 68 °C for 8 s, and a final extension at 68 °C for 10 min.

The purified PCR products were cloned into the pMD18-T vector (Takara, Dalian, China), transformed into *E. coli* DH-5α competent cells (Takara, Dalian, China), and sequenced (Sangon Biotech, Shanghai, China) in triplicate. The obtained nucleotide sequences were edited and assembled using the DNASTAR software (version 7.1, DNASTAR Inc., Madison, WI, USA). Open reading frames (ORFs) were predicted and annotated based on comparison with published PCV5 reference sequences. The putative stem-loop structure was identified according to the conserved circovirus genome organization and visualized for genomic characterization.

### 2.4. Construction of Phylogenetic Tree

Phylogenetic analyses were performed based on Rep and Cap amino acid sequences. Representative members of each species from the genera *Circovirus* and *Cyclovirus* were retrieved from GenBank (Table S3). The Rep dataset also included two closely related CRESS DNA virus sequences identified using the NCBI BLASTn web server (BLAST+ version 2.17.0), namely fur seal faeces-associated circular DNA virus and *Macochavirus* sp. The Cap dataset included representative circovirus and cyclovirus sequences and fur seal faeces-associated circular DNA virus (MK462122), while Macochavirus sp. (PX843015) was excluded due to the significant divergence observed in the Cap sequences.

The reference sequence set was selected to balance taxonomic representativeness and alignment reliability. Rep sequences were emphasized because Rep is generally more conserved than Cap among CRESS DNA viruses and is widely used for circovirus-related taxonomic and evolutionary comparisons. In contrast, Cap sequences are more variable and may be difficult to align reliably across highly divergent taxa. Therefore, the Rep- and Cap-based trees were interpreted together, with the Rep tree used as the principal framework and the Cap tree used as complementary evidence for sequence divergence.

Multiple sequence alignment was performed using MAFFT version 7.526 [26] with the E-INS-i strategy. The aligned sequences were imported into MEGA7, and the best-fit amino acid substitution model was selected based on the lowest BIC/AICc score. Maximum-likelihood (ML) phylogenetic trees were constructed using the selected models with 1000 bootstrap replicates. Partial deletion was applied with a site coverage cutoff of 80%. The resulting trees were used to assess the evolutionary relationship of PCV5-Henan2025-ZJQ01 with representative circoviruses, cycloviruses, and related CRESS DNA viruses.

### 2.5. Structural Prediction and Conserved Motif Identification

The three-dimensional structure of the Cap protein of PCV5-Henan2025-ZJQ01 was predicted using AlphaFold Server (https://alphafoldserver.com/). Linear B-cell epitopes of the Cap protein were predicted using the BepiPred 3.0 online tool (http://www.iedb.org/) with a default threshold of 0.5. The BioEdit version 7.2.5 was used to align Rep and putative Cap amino acid sequences of circovirus to show conserved motifs of PCV5-Henan2025-ZJQ01.

## 3. RESULTS

### 3.1. Epidemiologic Investigation of PCV5

**Figure 1:**
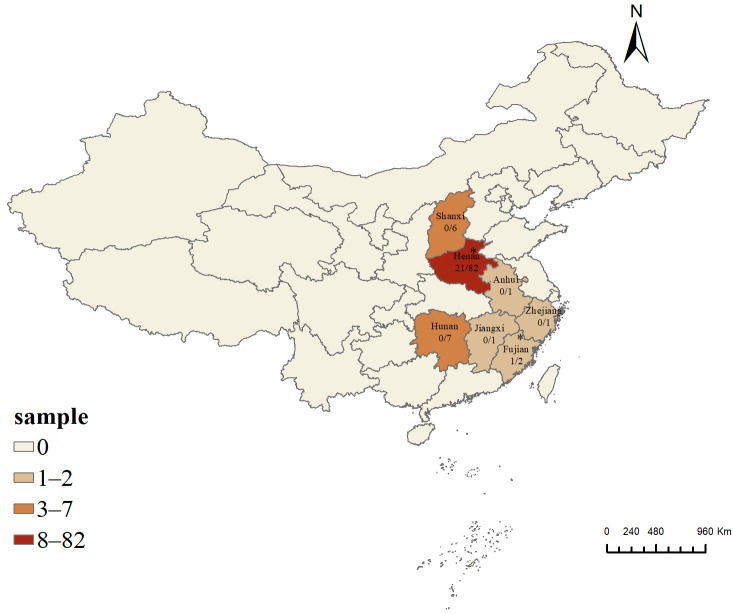
Geographical distribution of clinical samples tested for PCV5 in China in 2025. The color gradient indicates the number of samples tested in each province. * PCV5-positive samples were detected in Henan and Fujian provinces, whereas no PCV5-positive samples were detected in the other sampled provinces.

From February to December 2025, 22 of 100 diagnostic samples were tested positive for PCV5, yielding an overall detection rate of 22% (22/100) (Table 1). As shown in Figure 1, clinical samples were collected from seven provinces in China, and PCV5-positive samples were detected only in Henan and Fujian provinces among the sampled regions. According to sample type, PCV5 was detected in fecal samples (20/29, 68.97%), intestinal samples (1/36, 2.78%), and liver samples (1/5, 20.00%). No PCV5-positive results were detected in spleen (0/3), lung (0/1), blood (0/19), or rectal swab (0/7) samples. According to age group, PCV5 was detected in newborn piglets (3/9,33.33%), weaning pigs (16/59, 27.12%), and growers (3/12, 25.00%), whereas no PCV5-positive samples were detected in suckling piglets (0/5) or grow-finishing pigs (0/15).

In addition to PCV5, other swine viral pathogens were also detected in the 100 clinical samples, including PCV2, PCV3, PCV4, PEDV, PDCoV and PRRSV. The positive rates for PCV2, PCV3, PCV4, PEDV, PDCoV and PRRSV were 69% (69/100), 59% (59/100), 48% (48/100), 25% (25/100), 26% (26/100), and 3% (3/100), respectively (Table 1). Among the 22 PCV5-positive samples, only 4 samples were positive for PCV5 alone, whereas the remaining 18 samples were co-detected with at least one other swine viral pathogen (Table 2). Among these 18 co-detected PCV5-positive samples, PCV3 was the most frequently co-detected pathogen, followed by PCV2 and PEDV. The co-detection rates for PCV2, PCV3, PCV4, PEDV, PDCoV and PRRSV were 38.89% (7/18), 83.33% (15/18), 5.56% (1/18), 33.33% (6/18), 16.67% (3/18), and 0% (0/18), respectively. Multiple co-detection patterns were observed among PCV5, PCV2, PCV3, PCV4, PEDV, and PDCoV (Table 3). In one sample collected from a piglet with respiratory signs and diarrhea, PCV5, PCV2, PCV3, PCV4, and PDCoV were simultaneously detected. These findings indicate that PCV5 was frequently detected in field samples with other swine viral pathogens.

### 3.2. Genomic Structure of PCV5

Among the 22 PCV5-positive samples, the Cq values ranged from 26.66 to 30.90. Four samples with relatively low Cq values (26.66, 27.32, 28.95, and 29.45) were selected for complete genome amplification and sequencing. Although all four samples were subjected to PCR amplification, only two yielded complete genome sequences of sufficient quality for assembly and subsequent analyses, likely due to differences in viral load and template quality, whereas the remaining PCV5-positive samples were retained for sample-level detection and co-detection analysis. Sequence comparison showed that the two genomes were identical, but were collected from Henan and Fujian provinces, respectively. The obtained representative sequence was designated PCV5-Henan2025-ZJQ01 for subsequent genomic and phylogenetic analyses.

**Figure 2:**
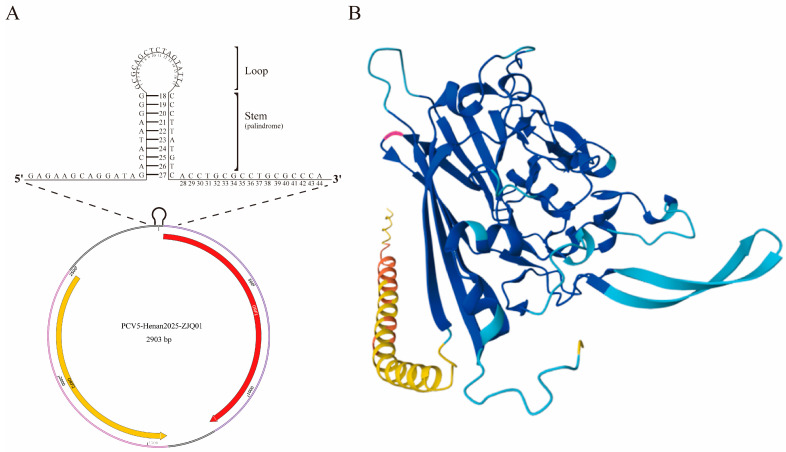
Genomic organization and predicted Cap structure of PCV5-Henan2025-ZJQ01. (**A**) Genomic organization and putative stem-loop structure of PCV5-Henan2025-ZJQ01. The predicted stem-loop structure contains a 10-bp palindromic stem and a 17-nt loop sequence. (**B**) Predicted three-dimensional structure of the putative Cap protein of PCV5-Henan2025-ZJQ01 generated by AlphaFold Server. Residues are coloured according to model confidence based on pLDDT scores: blue indicates very high confidence (pLDDT > 90), cyan indicates confident regions (70 < pLDDT < 90), yellow indicates low confidence (50 < pLDDT < 70), and orange indicates very low confidence (pLDDT < 50). The predicted template modelling score (pTM) was 0.81.

The sequence of PCV5-Henan2025-ZJQ01 was submitted to GenBank (accession number: PZ496079). The complete genome was 2903 nucleotides (nt) in length and exhibited a typical ambisense genomic organization characteristic of circoviruses (Figure 2A). Two major putative ORFs were identified, including a positive-sense ORF1 (1056 nt) encoding Cap and a negative-sense ORF2 (1182 nt) encoding Rep. The putative Cap and Rep proteins were further used for phylogenetic analysis. A putative stem-loop structure was identified in the intergenic region (Figure 2A). The predicted stem-loop structure contained a 10-bp palindromic stem and a 17-nt loop sequence (GCGCAGCTCTAGTATTA). The loop contained a TAGTATTA-like sequence, but did not completely match the conserved nonanucleotide motif (TAGTATTAC) commonly described in circoviruses [27]. In addition, the canonical rolling-circle replication-associated hexamer motif (CGGCAC) was not identified. Therefore, this region was considered a putative stem-loop structure rather than a confirmed replication origin.

### 3.3. Phylogenetic Analysis

PCV5-Henan2025-ZJQ01 strain shared 96.7% genomic nucleotide identity with PCV5 isolate PCV5-2904-CY (accession no. OR842903). The predicted Cap and Rep proteins of PCV5-Henan2025-ZJQ01 also showed high amino acid identity with those of PCV5-2904-CY, supporting its close relationship with previously reported PCV5 strains. The Cap protein of PCV5-Henan2025-ZJQ01 strain, consisting of 393 aa, shared 96.9% aa identity with that of PCV5 isolate PCV5-2904-CY, with 12 aa substitutions, at positions 26 (G to S), 47 (F to Y), 85 (T to K), 96 (R to N), 109 (A to S), 118 (S to T), 135 (G to N), 220 (Q to E), 223 (N to H), 237 (Q to N), 281 (A to T), and 390 (D to E).

**Figure 3:**
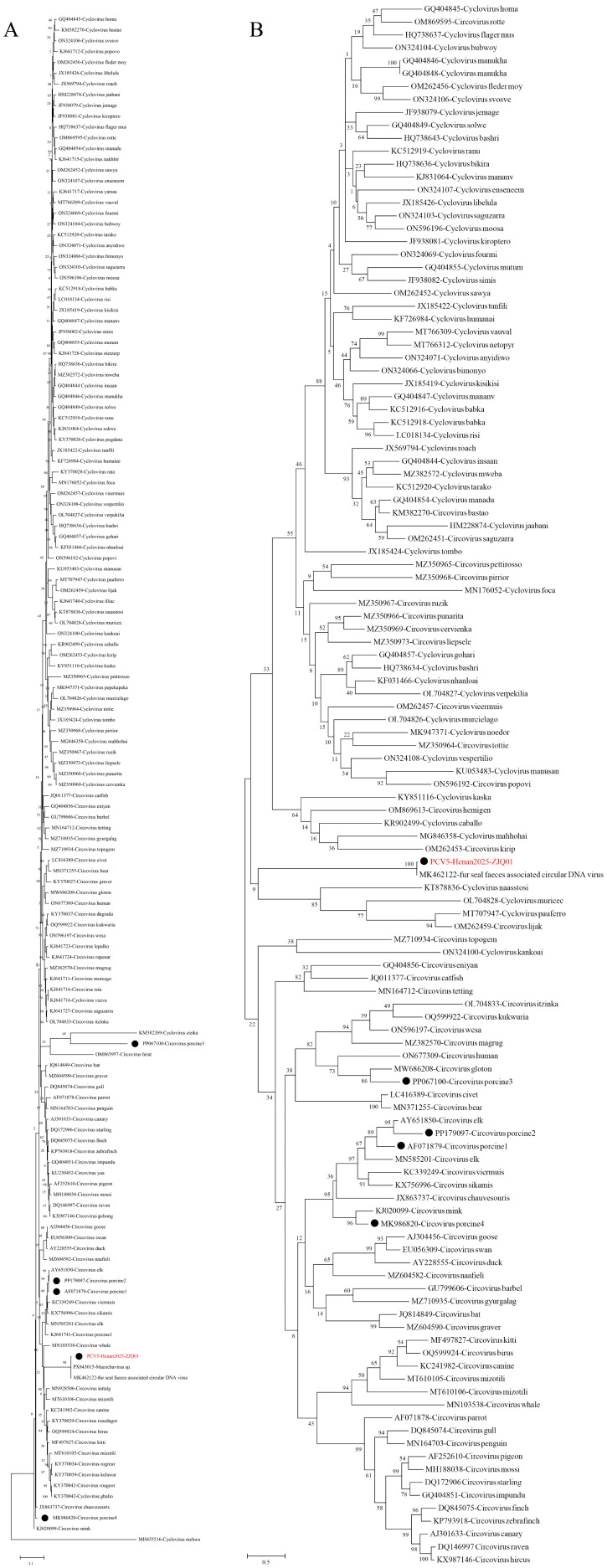
Maximum-likelihood (ML) analysis of PCV5-Henan2025-ZJQ01 based on Rep and Cap amino acid sequences. (**A**) The Rep amino acid tree was inferred using the ML method based on the LG + G substitution model. (**B**) The Cap amino acid tree was inferred using the ML method based on the rtREV + G + F substitution model. Porcine circovirus strains are indicated with black solid circles, and PCV5-Henan2025-ZJQ01 identified in this study is highlighted in red.

The ORF2 gene, encoding the Rep protein (351 amino acids), is oriented oppositely to the ORF encoding the Cap protein. The Rep protein of PCV5-Henan2025-ZJQ01 strain harboured 96% aa identity with that of PCV5 isolate PCV5-2904-CY and varied by 14 aa substitutions, at positions 51 (E to T), 163 (A to N), 164 (R to T), 167 (K to N), 168 (L to S), 171 (T to R), 208 (Y to H), 231 (S to T), 272 (A to S), 293 (T to S), 304 (S to A), 305 (S to A), 309 (I to V), and 313 (L to I). In the Rep-based phylogenetic tree, PCV5-Henan2025-ZJQ01 was clearly separated from most cyclovirus reference sequences and clustered with PCV5-related and circovirus-like CRESS DNA virus sequences, particularly fur seal faeces-associated circular DNA virus (MK462122) (Figure 3A).

For the Cap dataset, reliable alignment and phylogenetic reconstruction were more challenging because Cap sequences from circoviruses and cycloviruses were highly divergent (Figure 3B). PCV5-Henan2025-ZJQ01 did not cluster closely with most known PCVs in the Cap-based tree, suggesting that its Cap protein may be highly divergent. This divergence also supports the use of Rep-based phylogenetic analysis as the main evidence for evaluating the evolutionary relationship of PCV5-Henan2025-ZJQ01.

### 3.4. Structural Prediction and Motif Analysis of PCV5 Proteins

The Cap protein of PCV5-Henan2025-ZJQ01 showed low homology with most known circovirus Cap proteins. The predicted structure generated by AlphaFold Server suggested that the Cap protein may have a divergent structural profile compared with previously characterized circovirus Cap proteins (Figure 2B). In addition, no high-confidence linear B-cell epitope was predicted by BepiPred 3.0, with the highest predicted score of 0.258 below the recommended threshold. These results suggest that the Cap protein of PCV5-Henan2025-ZJQ01 may be highly divergent, and further experimental validation is required to determine its structural and antigenic properties.

**Figure 4:**
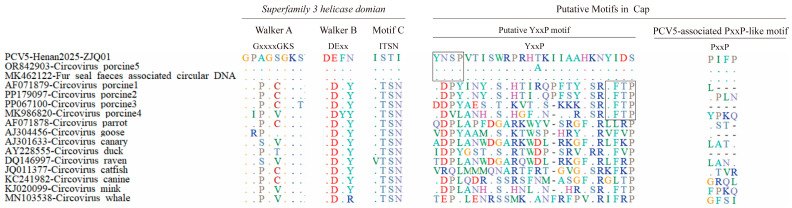
Sequence alignment and comparative motif analysis of Rep and putative Cap among representative circoviruses. Conserved helicase-associated motifs within the Reps, including Walker A (GxxxxGKS), Walker B (DExx), and motif C (ITSN), are shown on the left. Putative motifs identified in the aligned Cap region are shown on the right, including a putative YxxP motif and a putative PxxP-like motif observed in PCV5-related sequences. Amino acid residues identical to those of PCV5-Henan2025-ZJQ01 are represented by dots, and gaps are indicated by hyphens.

Alignment of the deduced amino acid sequences of Rep and putative Cap showed that PCV5-Henan2025-ZJQ01 retained several conserved motifs characteristic of circovirus-related proteins (Figure 4). In the Rep, conserved superfamily 3 helicase motifs were identified, including Walker A, Walker B, and motif C. These motifs were also present in representative circovirus reference sequences, including PCV1–PCV4 and selected non-porcine circoviruses, suggesting that the Rep of PCV5-Henan2025-ZJQ01 maintains the typical helicase-associated functional organization of circovirus Reps.

Comparative analysis of the putative Cap showed that PCV5-Henan2025-ZJQ01 contained a putative YxxP motif in the aligned region. Similar YxxP-like patterns were also observed in several representative circoviruses, although sequence variation was present in the surrounding residues. In addition, a putative PxxP-like motif was observed in PCV5-Henan2025-ZJQ01 and the PCV5 reference sequence, but was not consistently present in other representative circoviruses included in the alignment. These findings suggest that the putative Cap protein of PCV5 may contain distinctive motif features, although their functional significance requires further experimental validation.

## 4. Discussion

A previous study [22] reported that PCV5 was associated with respiratory, diarrheal, and reproductive disorders in pigs and provided multiple lines of evidence, including viral DNA detection in multiple tissues, histological lesions, virus isolation, electron microscopic visualization, and serological detection. In the present study, PCV5 was detected in diagnostic samples collected from clinically diseased pigs from commercial swine farms in China. PCV5 was detected in 22.0% (22/100) of the tested diagnostic samples. Because the samples were submitted during routine disease investigations rather than collected through a randomized prevalence survey, this value should be interpreted as a sample-level detection rate among clinically diseased pigs, rather than as farm-level or population-level prevalence.

PCV5-positive samples were mainly identified in fecal samples, with a small number of positive results detected in intestinal and liver samples. This sample-type distribution is broadly consistent with the previous report [22], in which PCV5 DNA was detected in intestinal tissues, intestinal lymph nodes, and feces. These observations suggest that fecal and intestinal samples may be useful sample types for PCV5 detection. However, the present study did not include systematic tissue distribution analysis, viral load comparison among organs, or matched clinically healthy controls. Therefore, the sample-type distribution observed here should be interpreted as preliminary molecular detection evidence rather than definitive evidence of tissue tropism.

The clinical significance of PCV5 detection should be interpreted cautiously. In this study, PCV5-positive samples were obtained from pigs showing diarrhea, respiratory signs, systemic disease, or wasting syndrome. However, these clinical signs are not specific for PCV5 infection and are commonly observed in field cases involving multiple swine pathogens. Only 4 of the 22 PCV5-positive samples were positive for PCV5 alone, whereas the remaining 18 samples were co-detected with at least one other pathogen, including PCV2, PCV3, PEDV, PDCoV, or PCV4. Therefore, the clinical signs observed in the sampled pigs cannot be attributed to PCV5 infection alone. The co-detection patterns observed in this study should be regarded as evidence of complex infection backgrounds in field diagnostic samples rather than evidence that PCV5 independently caused the observed clinical manifestations. Further studies including clinically healthy animals, virus isolation from additional cases, quantitative viral load analysis, and controlled infection experiments in pigs are needed to clarify the epidemiological and pathogenic significance of PCV5.

Two complete PCV5 genome sequences were obtained from PCV5-positive samples collected from Henan and Fujian provinces, respectively, and sequence comparison showed that the two genomes were identical. However, because only two complete genomes were available and animal movement or trade between regions cannot be excluded, these data are insufficient to infer regional circulation patterns, independent geographical spread, or the predominant circulating lineage of PCV5. More complete genome sequences from different provinces, farms, and sampling periods will be required to assess the genetic diversity, genetic stability, and evolutionary dynamics of PCV5 in pig populations.

The representative strain, PCV5-Henan2025-ZJQ01, had a genome length of 2903 nt and showed an ambisense genomic organization. Unlike classical porcine circoviruses PCV1–PCV4, in which ORF1 encodes Rep and ORF2 encodes Cap, PCV5-Henan2025-ZJQ01 contained a positive-sense ORF1 encoding Cap and a negative-sense ORF2 encoding Rep. This genomic arrangement is consistent with the reported characteristics of PCV5 [22] and further supports its distinction from previously recognized porcine circoviruses. The reversed arrangement of the major ORFs is noteworthy because gene orientation and intergenic architecture are important features in the comparative analysis of small circular DNA viruses. Such differences may reflect long-term divergence, recombination, or adaptation to a specific host or ecological niche, although the evolutionary mechanism underlying the PCV5 genome organization remains unknown. At present, the functional consequences of the Cap- and Rep-encoding ORFs in this orientation cannot be inferred from sequence data alone. Future studies examining replication intermediates, transcriptional profiles, and infectious virus systems will be needed to determine whether this genomic arrangement affects viral replication, gene expression, or host interaction.

The predicted intergenic stem-loop region of PCV5-Henan2025-ZJQ01 also showed atypical features. Although a 10-bp palindromic stem was identified, the 17-nt loop sequence did not completely match the conserved circovirus nonanucleotide motif, and the canonical rolling-circle replication-associated hexamer motif was not detected. Therefore, this region should be interpreted as a putative stem-loop structure rather than a confirmed replication origin. These atypical features may reflect the genetic distinctiveness of PCV5, but experimental validation is required to confirm the functional replication origin of this virus.

In the Rep-based phylogenetic analysis, fur seal faeces-associated circular DNA virus and Macochavirus sp. were included as additional CRESS DNA virus references because BLAST analysis showed relatively high similarity in conserved Rep regions. However, the relationship between these distant CRESS DNA virus sequences and PCV5-Henan2025-ZJQ01 should be interpreted cautiously. Their inclusion was intended to provide broader evolutionary context rather than to imply direct taxonomic assignment. Because CRESS DNA virus classification and evolutionary inference primarily rely on Rep, the Rep-based tree was considered more informative than the Cap-based tree, whereas the highly divergent Cap sequences were used as complementary evidence. In the Rep-based tree, PCV5-Henan2025-ZJQ01 clustered with PCV5-related CRESS DNA virus sequences and was separated from classical porcine circoviruses and most cyclovirus reference sequences. Conserved helicase-associated motifs, including Walker A, Walker B, and motif C, were also identified in the Rep protein, suggesting that PCV5-Henan2025-ZJQ01 retains the typical helicase-associated functional organization of circovirus-like Rep proteins.

In contrast to Rep, the Cap protein showed greater sequence divergence. Cap-based alignment and phylogenetic reconstruction were more challenging, likely because Cap proteins among circoviruses, cycloviruses, and related CRESS DNA viruses are highly variable. AlphaFold-based structural prediction suggested that the putative Cap protein of PCV5-Henan2025-ZJQ01 may have a divergent structural profile, and no high-confidence linear B-cell epitope was predicted by BepiPred 3.0. However, these bioinformatic predictions should be interpreted cautiously. Regions with low confidence scores in structural prediction may not accurately represent the true protein conformation, and predicted motifs or epitopes do not necessarily correspond to experimentally validated antigenic or functional regions. Therefore, the structural, antigenic, and biological properties of the PCV5 Cap protein require further experimental validation.

This study has several limitations. First, although samples were collected from multiple provinces, the sample size was limited and the sampling was based on diagnostic submissions from clinically diseased pigs. Therefore, the detection rate reported here should not be interpreted as farm-level or population-level prevalence, and farm-level prevalence, transmission patterns, or risk factors could not be inferred from the present dataset. Second, the frequent co-detection of other swine pathogens prevented us from determining the independent pathogenic role of PCV5. Third, only two identical complete genome sequences were obtained, which was insufficient to determine whether PCV5 circulating in the sampled regions exhibits low genetic diversity or whether this observation simply reflects the limited number of available genomes. Fourth, the predicted stem-loop structure, protein structure, linear B-cell epitopes, and amino-acid motifs were inferred using bioinformatic approaches and were not functionally validated. Additional complete genome sequences, larger-scale surveillance, clinically healthy control animals, serological investigations, virus isolation, controlled infection studies, and functional experiments will be required to clarify the prevalence, genetic diversity, tissue tropism, pathogenic potential, and immune relevance of PCV5.

Overall, the present work should be considered an initial molecular detection and genomic characterization study rather than a comprehensive epidemiological or pathogenicity investigation. The findings provide evidence that PCV5 was present in the tested diagnostic samples and that the representative genome PCV5-Henan2025-ZJQ01 is closely related to previously reported PCV5 sequences. These data expand the currently limited molecular information available for PCV5 and provide a basis for future surveillance, evolutionary analysis, and pathogenicity studies.

## 5. Conclusions

In this study, PCV5 was detected in 22.0% (22/100) of diagnostic samples collected from clinically diseased pigs in China. Two identical complete PCV5 genomes were obtained from samples collected in Henan and Fujian provinces, and the representative strain PCV5-Henan2025-ZJQ01 was further characterized. Genomic analysis showed that this strain possessed a 2903-nt genome, an atypical putative stem-loop region, and a distinct ORF arrangement compared with classical porcine circoviruses. Phylogenetic analysis indicated that PCV5-Henan2025-ZJQ01 was closely related to previously reported PCV5-related sequences but distinct from classical PCV1–PCV4. Co-detection analysis showed that most PCV5-positive samples were accompanied by other swine viral pathogens, indicating that the clinical significance of PCV5 requires cautious interpretation. These findings provide additional molecular and genomic evidence for PCV5 in Chinese swine diagnostic samples and support the need for continued surveillance and further experimental studies.

## Figures and Tables

**Table 1:** Detection of PCV5 and other swine viral pathogens in clinical samples collected from seven provinces in China.

Farm Name	Geographical Location	Collection Date	Sample Type	Clinical History	Day Age	No. of Samples	PCV5	PCV2	PCV3	PCV4	PEDV	PDCoV	PRRSV
Farm 1	Zhoukou, Henan Province	March 2025	Feces	Diarrhea	Weaning	6	6	2	3	0	3	0	0
Farm 2	Zhoukou, Henan Province	March 2025	Feces	Systemic	Newborn	2	2	0	2	0	1	0	0
Farm 3	Zhumadian, Henan Province	March 2025	Feces	Wasting	Grower	2	2	1	0	0	1	0	0
		March 2025	Feces	Wasting	Weaning	5	5	1	5	0	0	0	0
Farm 4	Zhoukou, Henan Province	March 2025	Feces	Systemic	Weaning	4	4	1	3	0	2	1	0
Farm 5	Pingdingshan, Henan Province	March 2025	Feces	Dyspnea, cough	Grower	1	1	1	0	0	1	0	0
Farm 6	Zhoukou, Henan Province	February 2025	Intestine	Dyspnea	Suckling	3	0	2	2	0	0	1	0
		April 2025	Intestine	Diarrhea	Suckling	2	0	1	0	1	0	0	0
Farm 7	Zhumadian, Henan Province	April 2025	Intestine	Systemic	Weaning	5	0	1	5	0	1	1	0
Farm 8	Zhumadian, Henan Province	April 2025	Intestine	Mild cough	Newborn	3	0	0	3	0	0	1	0
Farm 9	Zhumadian, Henan Province	February 2025	Intestine	Wasting	Weaning	3	0	2	3	0	0	0	0
Farm 10	Shangqiu, Henan Province	April 2025	Intestine	Systemic	Grower	4	0	4	2	2	0	0	0
Farm 11	Shangqiu, Henan Province	April 2025	Intestine	Systemic	Grower	3	0	3	3	1	0	2	0
Farm 12	Shangqiu, Henan Province	March 2025	Intestine	Dyspnea	Weaning	5	0	5	3	0	0	0	0
Farm 13	Anqing, Anhui Province	March 2025	Liver	Systemic	Weaning	1	0	1	1	1	0	1	1
Farm 14	Jincheng, Shanxi Province	March 2025	Spleen	Dyspnea/Diarrhea	Weaning	1	0	1	0	1	0	1	0
		March 2025	Lung	Cough/Diarrhea	Weaning	1	0	1	1	1	1	1	0
Farm 15	Xinyang, Henan Province	March 2025	Spleen	Mild tachypnea	Grower	1	0	1	0	1	0	1	0
Farm 16	Huixian, Henan Province	March 2025	Liver	Cough/Diarrhea	Newborn	1	1	1	1	1	0	1	0
Farm 17	Nanyang, Henan Province	March 2025	Liver	Dyspnea/Diarrhea	Newborn	1	0	1	1	1	0	0	0
Farm 18	Lishui, Zhejiang Province	March 2025	Liver	Cough	Grower	1	0	1	1	0	0	1	0
Farm 19	Ganzhou, Jiangxi Province	March 2025	Liver	Systemic	Newborn	1	0	1	1	1	1	1	1
Farm 20	Xuchang, Henan Province	March 2025	Spleen	Dyspnea	Newborn	1	0	1	1	1	0	1	0
Farm 21	Pingdingshan, Henan Province	December 2025	Blood	Systemic	Grow-finish	15	0	15	8	15	1	0	1
Farm 22	Yuncheng, Shanxi Province	August 2025	Blood	Dyspnea	Weaning	4	0	4	4	4	0	0	0
Farm 23	Luoyang, Henan Province	December 2025	Feces	Cough/Dyspnea	Weaning	9	0	9	2	9	4	1	0
Farm 24	Luoyang, Henan Province	December 2025	Intestine	Diarrhea	Weaning	3	0	3	2	1	0	3	0
Farm 25	Zhumadian, Henan Province	December 2025	Intestine	Diarrhea	Weaning	3	0	3	0	2	3	3	0
Farm 26	Changsha, Hunan Province	April 2025	Rectal swab	Diarrhea	Weaning	7	0	7	1	5	4	3	0
Farm 27	Sanming, Fujian Province	April 2025	Intestine	Diarrhea	Weaning	2	1	0	1	0	2	2	0
Total						100	22/100; 22%	69/100; 69%	59/100 59%	48/100 48%	25/100 25%	26/100 26%	3/100 3%

**Table 2:** PCV5-positive sample groups and co-detected pathogens.

Province	Farm Name	Sample Type	Clinical History	Day Age	PCV5-Positive/Tested	Co-Detected Pathogens	Co-Detection Pattern
Henan	Farm 1	Feces	Diarrhea	Weaning	6/6	PCV2, PCV3, PEDV	PCV5 + PCV2/PCV3/PEDV
Henan	Farm 2	Feces	Systemic	Newborn	2/2	PCV3, PEDV	PCV5 + PCV3/PEDV
Henan	Farm 3	Feces	Wasting	Grower	2/2	PCV2, PEDV	PCV5 + PCV2/PEDV
Henan	Farm 3	Feces	Wasting	Weaning	5/5	PCV2, PCV3	PCV5 + PCV2/PCV3
Henan	Farm 4	Feces	Systemic	Weaning	4/4	PCV2, PCV3, PEDV, PDCoV	PCV5 + multiple pathogens
Henan	Farm 5	Feces	Respiratory	Grower	1/1	PCV2, PEDV	PCV5 + PCV2/PEDV
Henan	Farm 16	Liver	Respiratory/diarrhea	Newborn	1/1	PCV2, PCV3, PCV4, PDCoV	PCV5 + multiple pathogens
Fujian	Farm 27	Intestine	Diarrhea	Weaning	1/2	PCV3, PEDV, PDCoV	PCV5 + PCV3/PEDV/PDCoV

**Table 3:** Co-detected pathogens in PCV5-positive samples.

Co-Detection Pattern Among PCV5-Positive Samples	No. of Samples
PCV5 + PCV3	7
PCV5 + PEDV	1
PCV5 + PCV2 + PCV3	3
PCV5 + PCV2 + PEDV	2
PCV5 + PCV3 + PEDV	1
PCV5 + PCV3 + PDCoV	1
PCV5 + PCV2 +PCV3 + PEDV	1
PCV5 + PCV3 + PEDV + PDCoV	1
PCV5 + PCV2 +PCV3 + PCV4 + PDCoV	1

## Data Availability

The data presented in this study are openly available in GenBank under accession number PZ496079.

## Supplementary Materials

The following supporting information can be downloaded at: https://www.mdpi.com/article/10.3390/vetsci13070614/s1, Table S1. List of primer sequences for qPCR in this study. Table S2. List of primer sequences used in this study. Table S3. List of reference sequences for phylogenetic inference of viruses in the genera *Circovirus* and *Cyclovirus*.
