# Supplementary material for: First Molecular Detection and Genetic Characterization of Porcine Circovirus 5 in Diagnostic Swine Samples from China
Source: Vet Sci. 2026 Jun 25;13(7):614. doi: 10.3390/vetsci13070614 (PMC13418592; doi:10.3390/vetsci13070614)
Supplement: Supplementary file 1 [file vetsci-13-00614-s001.zip › vetsci-4368029-supplementary.pdf]

Supplementary Information

First molecular detection and genetic characterization of porcine circovirus 5 in Henan Province, China

Jia-qi Zhang[1], Jia-xin Li[2], Hui-lin Qu[1], Yu-jie Miao[1], Xi-meng Chen[1], Lan-Lan Zheng[1.3.4], Yi-lei Li[1.3.4*], Hong-ying Chen[1.3.4*], Shi-Jie Ma[1.3.4*]

[1]College of Veterinary Medicine, Henan Agricultural University, Zhengdong New District Longzi Lake 15#, Zhengzhou 450046, PR China.

[2]Faculty of Science, University of Melbourne, Parkville, VIC 3010, Australia.

[3]Ministry of Education Key Laboratory for Animal Pathogens and Biosafety, Zhengzhou 450046, PR China.

[4]Henan Province Key Laboratory for Animal Food Pathogens Surveillance, Zhengzhou 450046, PR China

[†] Jia-qi Zhang, Jia-xin Li and Hui-lin Qu contributed equally to this work.

[*]**Corresponding author**: Hong-ying Chen, E-mail: chhy927@163.com, Yi-lei Li E-mail: 13526583021@163.com and Shi-Jie Ma, E-mail: participate2006@126.com. Mailing address: College of Veterinary Medicine, Henan Agricultural University, Zhengdong New District Longzi Lake[#]15, 450046 Zhengzhou, Henan Province, People's Republic of China. Tel: +86 371 55369208; fax: +86 371 55369208

Table Legends

Table S1. List of primer sequences for qPCR in this study.

Table S2. List of primer sequences used in this study.

Table S3. List of reference sequences for phylogenetic inference of viruses in the genera *Circovirus* and *Cyclovirus*.

Table S1 List of primer sequences for qPCR in this study.

Organism	Nucleotide sequence (5'-3')
PCV5	F: ACGGTGCTAGCAAAGTGTGA
	R: GCCAACTACCAAGCCGAAGA
PRRSV	F: AAACCAGTCCAGAGGCAAGC
	R: GCAAACTAAACTCCACAGTGTAA
PDCoV	F: CGTTAACCTCTTCTCACCACTT
	R: GCTGAGAGTCTGGTTGGTTATT
PEDV	F: CGCAAAGACTGAACCCACTAATTT
	R: TTGCCTCTGTTGTTACTTGGAGAT
Porcine circovirus 2	F: AGTCTCAGCCACAGCTGATT
	R: TCCTCCCGCCATACCAT
	Probe: Cy5-AGCCCTTCTCCTACCACTCCCGCT-BHQ2
Porcine circovirus 3	F: CGGATTCTGACGGAGACG
	R: TCACGCGGTTTACCCAACCC
	Probe: FAM-GCTATGGGCGGGGTTTGCGT-TAMRA
Porcine circovirus 4	F: GTCCACACCTGCACAAAGTT
	R: CCTCCACTTCCAGCCTAACA
	Probe: Texas Red-AGGTCCTGGTCCGCCATGCT-BHQ2

Note: The primer of PCV5 was taken from previous publication (Liu, et al. 2025).
The primer of PRRSV was taken from previous publication (Li, et al. 2024).
The primer of PDCoV and PEDV were from taken from previous publication (Zhang, et al. 2022).
The primer of PCV2, PCV3 and PCV4 was taken from previous publication (Zou, et al. 2022).

Liu, X., et al. 2025 Identification and biochemical characterization of a novel porcine circovirus associated with porcine respiratory and diarrheal diseases. Microbiol Spectr 13(11):e0229925.

Li, W., et al. 2024 PRRSV GP5 inhibits the antivirus effects of chaperone-mediated autophagy by targeting LAMP2A. mBio 15(8):e0053224.

Zhang, H., et al. 2022 Co-infection of porcine epidemic diarrhoea virus and porcine deltacoronavirus enhances the disease severity in piglets. Transbound Emerg Dis 69(4):1715-1726.

Zou, J., et al. 2022 Development of a TaqMan-Probe-Based Multiplex Real-Time PCR for the Simultaneous Detection of Porcine Circovirus 2, 3, and 4 in East China from 2020 to 2022. Vet Sci 10(1).

Table S2 List of primer sequences used in this study

Name	Nucleotide sequence (5’-3’)
PCV5-1	F: TTTAAGTATGGATTTATGAAGTGTTGT
	R: AAGCACCATAACCCTTACCAAGTTG
PCV5-2	F: TATTGGATGGGTCCATGTATCCGTCT
	R: GTCAGATATTTGTCAATCGGAAAAC
PCV5-3	F: TACTTCTCAAGTCCACTAGAGTATCTT
	R: ACCACTTACAGTTAAACCTGAGGACTT

Note: The primer of PCV5 was taken from previous publication (Liu, et al. 2025).

Liu, X., et al. 2025 Identification and biochemical characterization of a novel porcine circovirus associated with porcine respiratory and diarrheal diseases. Microbiol Spectr 13(11):e0229925.

Table S3 List of reference sequences for phylogenetic inference of viruses in the genera *Circovirus* and *Cyclovirus*.

GenBank accession number	Circovirus	GenBank accession number	Cyclovirus
AF071878	Circovirus parrot	GQ404844	Cyclovirus insaan
AF071879	Circovirus porcine1	GQ404845	Cyclovirus homa
AF252610	Circovirus pigeon	GQ404846	Cyclovirus manukha
AJ301633	Circovirus canary	GQ404847	Cyclovirus mananv
AJ304456	Circovirus goose	GQ404849	Cyclovirus solwe
AY228555	Circovirus duck	GQ404854	Cyclovirus manadu
AY651850	Circovirus elk	GQ404855	Cyclovirus mutum
DQ146997	Circovirus raven	GQ404857	Cyclovirus gohari
DQ172906	Circovirus starling	HM228874	Cyclovirus jaabani
DQ845074	Circovirus gull	HQ738634	Cyclovirus bashri
DQ845075	Circovirus finch	HQ738636	Cyclovirus bikira
EU056309	Circovirus swan	HQ738637	Cyclovirus_flager_mus
GQ404851	Circovirus impundu	JF938079	Cyclovirus jemage
GQ404856	Circovirus eniyan	JF938081	Cyclovirus kiroptero
GU799606	Circovirus barbel	JX185419	Cyclovirus kisikisi
JQ011377	Circovirus catfish	JX185422	Cyclovirus tunfili
JQ814849	Circovirus bianfu	JX185424	Cyclovirus tombo
JX863737	Circovirus chauvesouris	JX185426	Cyclovirus libelula
KC241982	Circovirus canine	JX569794	Cyclovirus roach
KC339249	Circovirus viermuis	KC512918	Cyclovirus_babka
KJ020099	Circovirus mink	KC512919	Cyclovirus ranu
KP793918	Circovirus zebrafinch	KC512920	Cyclovirus tarako
PP067100	Circovirus porcine3	KF031466	Cyclovirus nhanloai
KU230452	Circovrus yaa	KF726984	Cyclovirus_humanai
KX756996	Circovirus sikamis	KJ831064	Cyclovirus manukha
KX987146	Circovirus pchong	KM382270	Cyclovirus bastao
LC416389	Circovirus civet	KR902499	Cyclovirus caballo
MF497827	Circovirus kitti	KT878836	Cyclovirus naaasstosi
MH188038	Circovirus mossi	KU053483	Cyclovirus manusan
MK986820	Circovirus porcine4	KY851116	Cyclovirus kaska
MN103538	Circovirus whale	LC018134	Cyclovirus risi
MN164703	Circovirus penguin	MG846358	Cyclovirus mahhohai
MN164712	Circovirus tetting	MK947371	Cyclovirus noedor
MN371255	Circovirus bear	MN176052	Cyclovirus_foca
MN585201	Circovirus elk	MT707947	Cyclovirus_pauferro
MT610105	Circovirus mizotili	MT766309	Cyclovirus_vauval
MT610106	Circovirus mizotili	MZ350964	Cyclovirus tottie
MW686208	Circovirus gloton	MZ350965	Cyclovirus pettirosso
MZ350966	Circovirus_punarita	MZ350967	Cyclovirus ruzik
MZ382570	Circovirus magrug	MZ350968	Cyclovirus pirrior

MZ604582	Circovirus naafieli	MZ350969	Cyclovirus cervienka
MZ604590	Circovirus graver	MZ350973	Cyclovirus liepsele
MZ710934	Circovirus topogem	MZ382572	Cyclovirus mweba
MZ710935	Circovirus gyurgalag	OL704826	Cyclovirus murcielago
OL704833	Circovirus itzinka	OL704827	Cyclovirus verpekilia
ON596197	Circovirus wesa	OL704828	Cyclovirus muricec
ON677309	Circovirus human	OM262452	Cyclovirus_sawya
OQ599922	Circovirus kukwuria	OM262453	Cyclovirus kirip
OQ599924	Circovirus birus	OM262456	Cyclovirus_fleder_moy
PP179097	Circovirus porcine2	OM262457	Cyclovirus vieermuis
KJ641711*	Circovirus morcego	OM262459	Cyclovirus lijak
KJ641716*	Circovirus tzia	OM869595	Cyclovirus rotte
KJ641723*	Circovirus lepalko	ON324066	Cyclovirus bimonyo
KJ641724*	Circovirus_rapenat	ON324069	Cyclovirus fourmi
KJ641727*	Circovirus saguzarra	ON324071	Cyclovirus anyidiwo
KM382269*	Cyclovirus_zizika	ON324100	Cyclovirus kankoai
KY370027*	Circovirus graver	ON324103	Cyclovirus saguzarra
KY370029*	Circovirus rosedagor	ON324104	Cyclovirus bubwoy
KY370034*	Circovirus rogreur	ON324106	Cyclovirus svosve
KY370037*	Circovirus dagruda	ON324107	Cyclovirus enseneem
KY370039*	Circovirus kelawar	ON324108	Cyclovirus vespertilio
KY370042*	Circovirus rougeor	ON596192	Cyclovirus popovi
MN928506*	Circovirus iattalg	ON596196	Cyclovirus moosa
OM865957*	Circovirus_hirat	JF938082*	Cyclovirus simis
OM262451+	Circovirus saguzaarra	KJ641712*	Cyclovirus popovo
OM869613+	Circovirus hemigen	KJ641715*	Cyclovirus nakhhir
		KJ641717*	Cyclovirus yarasa
		KJ641728*	Cyclovirus nietzerp
		KJ641740*	Cyclovirus illiac
		KJ641716*	Cyclovirus vazva
		KY370042*	Cyclovirus gbalio
		KY370026*	Cyclovirus pogdana
		KY370028*	Cyclovirus rata
		MH455516*	Cyclovirus_mchwa
		GQ404848+	Cyclovirus manukha
		HQ738643+	Cyclovirus bashri
		KC512916+	Cyclovirus babka
		MT766312+	Cyclovirus netopyr

Note: An asterisk (*) indicates that the amino acid sequence corresponding to the accession number consists solely of the Rep database, while a plus sign (+) indicates that the amino acid sequence consists solely of the Cap database.

The reference amino acid sequences were taken from previous publication (Varsani, et al. 2024).

Varsani, A., et al. 2024 2024 taxonomy update for the family Circoviridae. Arch Virol 169(9):176.
